# Supplementary material for: Vulnerability of amphibians to global warming
Source: Nature. 2025 Mar 5;639(8056):954–61. doi: 10.1038/s41586-025-08665-0 (PMC11946914; doi:10.1038/s41586-025-08665-0)
Supplement: Supplementary file 1 — Supplementary Tables 1–5. [file 41586_2025_8665_MOESM1_ESM.pdf]

nature portfolio

https://doi.org/10.1038/s41586-025-08665-0

Supplementary information

Vulnerability of amphibians to global warming

In the format provided by the
authors and unedited

Vulnerability of amphibians to global warming

Patrice Pottier[1,2*], Michael R. Kearney[3], Nicholas C. Wu[4], Alex R. Gunderson[5], Julie E. Rej[5], A. Nayelli Rivera-Villanueva[6,7], Pietro Pollo[1], Samantha Burke[1], Szymon M. Drobniak[1,8+], and Shinichi Nakagawa[1,9+]

[1] Evolution & Ecology Research Centre, School of Biological, Earth and Environmental Sciences, University of New South Wales, Sydney, New South Wales, Australia.

[2] Division of Ecology and Evolution, Research School of Biology, The Australian National University, Canberra, Australian Capital Territory, Australia

[3] School of BioSciences, The University of Melbourne, Melbourne, Victoria, Australia

[4] Hawkesbury Institute for the Environment, Western Sydney University, Richmond, New South Wales, Australia

[5] Department of Ecology and Evolutionary Biology, Tulane University, New Orleans, Louisiana, USA

[6] Centro Interdisciplinario de Investigación para el Desarrollo Integral Regional Unidad Durango (CIIDIR), Instituto Politécnico Nacional, Durango, México

[7] Laboratorio de Biología de la Conservación y Desarrollo Sostenible de la Facultad de Ciencias Biológicas, Universidad Autónoma de Nuevo León, Monterrey, México

[8] Institute of Environmental Sciences, Jagiellonian University, Kraków, Poland.

[9] Department of Biological Sciences, University of Alberta, Edmonton, Alberta, Canada.

[*]Corresponding author

[+]These authors supervised the work equally

Corresponding author: Patrice Pottier (p.pottier@unsw.edu.au)

Table of contents

Supplementary Tables 3
Table S1 3
Table S2 4
Table S3 5
Table S4 6
Table S5 7

Supplementary tables

Table S1 | Statistical model estimates for thermal safety margins calculated for local species occurrences and assemblages Model estimates for each microhabitat (terrestrial, arboreal, aquatic) and each climatic scenario (current, +2°C, or +4°C of global warming above pre-industrial levels) are depicted. No contrast structure was used in the presented models. mean: mean model estimate; CI.lb: lower bound of the 95% confidence interval; CI.ub: upper bound of the 95% confidence interval; p: p-value; k_{sp}: number of species; k_{obs}: number of observations; Var_{sp}: variance explained by differences between species; Var_{phy}: variance explained by shared evolutionary history; Var_{obs}: residual variance.

	Local species patterns in thermal safety margin								
	**mean**	**CI.lb**	**CI.ub**	**p**	**k_{sp}**	**k_{obs}**	**Var_{sp}**	**Var_{phy}**	**Var_{obs}**
Terrestrial (current)	11.694	8.856	14.428	<0.001	5177	203853			
Terrestrial (+2°C)	10.914	8.025	13.594	<0.001	5177	203853			
Terrestrial (+4°C)	9.409	6.530	12.090	<0.001	5177	203853			
Arboreal (current)	12.235	9.402	14.960	<0.001	1771	56210			
Arboreal (+2°C)	11.517	8.660	14.236	<0.001	1771	56210	1.295	11.960	1.828
Arboreal (+4°C)	10.073	7.229	12.797	<0.001	1771	56210			
Aquatic (current)	13.598	10.708	16.276	<0.001	5203	204808			
Aquatic (+2°C)	12.827	8.796	14.361	<0.001	5203	204808			
Aquatic (+4°C)	11.682	8.796	14.361	<0.001	5203	204808			

	Assemblage-level patterns in thermal safety margin					
	**mean**	**CI.lb**	**CI.ub**	**p**	**k_{obs}**	**Var_{obs}**
Terrestrial (current)	15.279	15.208	15.330	<0.001	14090	
Terrestrial (+2°C)	14.328	14.279	14.396	<0.001	14090	
Terrestrial (+4°C)	12.602	12.542	12.657	<0.001	14090	
Arboreal (current)	14.279	14.191	14.381	<0.001	6614	11.06
Arboreal (+2°C)	13.393	13.298	13.478	<0.001	6614	
Arboreal (+4°C)	11.746	11.666	11.830	<0.001	6614	
Aquatic (current)	17.408	17.352	17.471	<0.001	14091	
Aquatic (+2°C)	16.528	16.468	16.581	<0.001	14091	
Aquatic (+4°C)	15.287	15.225	15.346	<0.001	14091	

Table S2 | Statistical model estimates for overheating risk and the number of overheating events. Model estimates for each microhabitat (terrestrial, arboreal) and each climatic scenario (current, +2°C, or +4°C of global warming above pre-industrial levels) are depicted. The estimated number of overheating events in species predicted to experience at least one overheating event (i.e., overheating species) are also depicted. Model estimates for aquatic microhabitats are not displayed because no species was predicted to experience overheating events in this microhabitat. No contrast structure was used in the presented models. mean: mean model estimate; CI.lb: lower bound of the 95% confidence interval; CI.ub: upper bound of the 95% confidence interval; p: p-value; k_{sp}: number of genera; k_{sp}: number of species; k_{obs}: number of observations; Var_{genus}: variance explained by differences between genera; Var_{sp}: variance explained by differences between species; Var_{obs}: residual variance.

Overheating risk

	mean	CI.lb	CI.ub	p	k_{genus}	k_{sp}	k_{obs}	Var_{genus}	Var_{sp}
Terrestrial (current)	9.98×10^{-7}	5.60×10^{-7}	1.78×10^{-6}	<0.001	464	5177	203853		
Terrestrial (+2°C)	1.93×10^{-6}	1.09×10^{-6}	3.43×10^{-6}	<0.001	464	5177	203853		
Terrestrial (+4°C)	9.09×10^{-6}	5.13×10^{-6}	1.61×10^{-5}	<0.001	464	5177	203853	0.306	69.653
Arboreal (current)	4.77×10^{-7}	2.58×10^{-7}	8.80×10^{-7}	<0.001	174	1771	56210		
Arboreal (+2°C)	9.78×10^{-7}	5.45×10^{-7}	1.75×10^{-6}	<0.001	174	1771	56210		
Arboreal (+4°C)	3.72×10^{-6}	2.08×10^{-6}	6.67×10^{-6}	<0.001	174	1771	56210		

Number of overheating events (all species)

	mean	CI.lb	CI.ub	p	k_{genus}	k_{sp}	k_{obs}	Var_{genus}	Var_{sp}
Terrestrial (current)	0.014	0.001	0.080	<0.001	464	5177	203853		
Terrestrial (+2°C)	0.025	0.002	0.127	<0.001	464	5177	203853		
Terrestrial (+4°C)	0.153	0.046	0.460	<0.001	464	5177	203853	0.110	52.500
Arboreal (current)	0.008	0.001	0.043	<0.001	174	1771	56210		
Arboreal (+2°C)	0.015	0.001	0.083	<0.001	174	1771	56210		
Arboreal (+4°C)	0.076	0.012	0.230	<0.001	174	1771	56210		

Number of overheating events (among overheating species)

	mean	CI.lb	CI.ub	p	k_{genus}	k_{sp}	k_{obs}	Var_{genus}	Var_{sp}	Var_{obs}
Terrestrial (current)	2.155	0.239	5.264	<0.001	38	104	836			
Terrestrial (+2°C)	2.576	0.410	5.857	<0.001	61	168	1424			
Terrestrial (+4°C)	6.747	3.136	11.385	<0.001	118	391	4248	0.253	0.187	0.310
Arboreal (current)	1.621	0.026	4.429	<0.001	4	13	152			
Arboreal (+2°C)	1.956	0.113	4.973	<0.001	5	16	283			
Arboreal (+4°C)	5.084	1.806	9.387	<0.001	17	56	748			

Table S3 | Statistical model estimates for the number of species predicted to experience overheating events. Model estimates for each microhabitat (terrestrial, arboreal) and each climatic scenario (current, +2°C, or +4°C of global warming above pre-industrial levels) are depicted. The estimated number of species overheating in assemblages containing at least one species predicted to experience at least one overheating event (i.e., overheating assemblages) are also depicted. Model estimates for aquatic microhabitats are not displayed because no species was predicted to experience overheating events in this microhabitat. No contrast structure was used in the presented models. mean: mean model estimate; CI.lb: lower bound of the 95% confidence interval; CI.ub: upper bound of the 95% confidence interval; p: p-value; k_{obs}: number of observations; Var_{obs}: residual variance.

	Number of species overheating (all assemblages)					
	**mean**	**CI.lb**	**CI.ub**	**p**	**k_{obs}**	**Var_{obs}**
Terrestrial (current)	0.056	0.016	0.118	<0.001	14090	55.47
Terrestrial (+2°C)	0.096	0.029	0.199	<0.001	14090	
Terrestrial (+4°C)	0.288	0.083	0.604	<0.001	14090	
Arboreal (current)	0.021	0.002	0.054	<0.001	6614	
Arboreal (+2°C)	0.040	0.006	0.094	<0.001	6614	
Arboreal (+4°C)	0.107	0.021	0.243	<0.001	6614	
	Number of species overheating (among overheating assemblages)					
	**mean**	**CI.lb**	**CI.ub**	**p**	**k_{obs}**	**Var_{obs}**
Terrestrial (current)	3.185	0.601	6.883	<0.001	253	0.601
Terrestrial (+2°C)	3.228	0.678	6.810	<0.001	426	
Terrestrial (+4°C)	3.084	0.617	6.557	<0.001	1328	
Arboreal (current)	1.930	0.054	5.054	<0.001	74	
Arboreal (+2°C)	2.445	0.189	5.649	<0.001	111	
Arboreal (+4°C)	2.509	0.312	5.692	<0.001	285	

Table S4 | Statistical model estimates for the proportion of species predicted to experience overheating events. Model estimates for each microhabitat (terrestrial, arboreal) and each climatic scenario (current, +2°C, or +4°C of global warming above pre-industrial levels) are depicted. The estimated proportion of species overheating in assemblages containing at least one species predicted to experience at least one overheating event (i.e., overheating assemblages) are also depicted. Model estimates for aquatic microhabitats are not displayed because no species was predicted to experience overheating events in this microhabitat. No contrast structure was used in the presented models. mean: mean model estimate; CI.lb: lower bound of the 95% confidence interval; CI.ub: upper bound of the 95% confidence interval; p: p-value; k_{obs}: number of observations; Var_{obs}: residual variance.

	Proportion of species overheating (all assemblages)					
	**mean**	**CI.lb**	**CI.ub**	**p**	k_{obs}	Var_{obs}
Terrestrial (current)	1.22×10^{-5}	8.96×10^{-6}	1.66×10^{-5}	<0.001	14090	42.26
Terrestrial (+2°C)	2.09×10^{-5}	1.60×10^{-5}	2.72×10^{-5}	<0.001	14090	
Terrestrial (+4°C)	8.13×10^{-5}	6.60×10^{-5}	1.00×10^{-4}	<0.001	14090	
Arboreal (current)	1.19×10^{-5}	7.07×10^{-6}	2.02×10^{-5}	<0.001	6614	
Arboreal (+2°C)	1.86×10^{-5}	1.19×10^{-5}	2.89×10^{-5}	<0.001	6614	
Arboreal (+4°C)	4.99×10^{-5}	3.62×10^{-5}	6.87×10^{-5}	<0.001	6614	

	Proportion of species overheating (among overheating assemblages)					
	**mean**	**CI.lb**	**CI.ub**	**p**	k_{obs}	Var_{obs}
Terrestrial (current)	0.053	0.046	0.061	<0.001	253	1.019
Terrestrial (+2°C)	0.058	0.052	0.065	<0.001	426	
Terrestrial (+4°C)	0.094	0.088	0.100	<0.001	1328	
Arboreal (current)	0.038	0.029	0.050	<0.001	74	
Arboreal (+2°C)	0.054	0.043	0.067	<0.001	111	
Arboreal (+4°C)	0.061	0.053	0.070	<0.001	285	

Table S5 | Statistical model estimates for the association between the number of overheating events and thermal safety margins. Model estimates for each microhabitat (terrestrial, arboreal) and each climatic scenario (current, +2°C, or +4°C of global warming above pre-industrial levels) are depicted. Model estimates for aquatic microhabitats are not displayed because no species was predicted to experience overheating events in this microhabitat. All model estimates are on the log scale. Separate models were fitted for each microhabitat and climatic scenario. mean: mean model estimate; se: standard error; p: p-value; k_{sp}: number of genera; k_{sp}: number of species; k_{obs}: number of observations; Var_{genus}: variance explained by differences between genera; Var_{sp}: variance explained by differences between species; Var_{obs}: residual variance.

	mean	se	p	k_{genus}	k_{sp}	k_{obs}	Var_{genus}	Var_{sp}	Var_{obs}
Terrestrial (current)									
Intercept	3.723	0.390	<0.001	464	5177	203853	5.850	3.346	0.116
Slope (TSM)	-1.201	0.031	<0.001						
Terrestrial (+2°C)									
Intercept	6.318	0.310	<0.001	464	5177	203853	5.272	2.380	0.078
Slope (TSM)	-1.452	0.027	<0.001						
Terrestrial (+4°C)									
Intercept	7.611	0.171	<0.001	464	5177	203853	2.954	1.025	0.248
Slope (TSM)	-1.616	0.015	<0.001						
Arboreal (current)									
Intercept	4.929	1.091	<0.001	174	1771	56210	0.001	15.190	0.001
Slope (TSM)	-1.511	0.094	<0.001						
Arboreal (+2°C)									
Intercept	7.836	0.836	<0.001	174	1771	56210	4.359	2.358	0.001
Slope (TSM)	-1.739	0.080	<0.001						
Arboreal (+4°C)									
Intercept	10.093	0.587	<0.001	174	1771	56210	8.789	0.917	0.001
Slope (TSM)	-2.085	0.039	<0.001						
