# Supplementary material for: Vulnerability of amphibians to global warming
Source: Nature. 2025 Mar 5;639(8056):954–61. doi: 10.1038/s41586-025-08665-0 (PMC11946914; doi:10.1038/s41586-025-08665-0)
Supplement: Supplementary file 2 — Reporting Summary [file 41586_2025_8665_MOESM2_ESM.pdf]

nature portfolio

Corresponding author(s): Patrice Pottier

Last updated by author(s): 16/12/2024

Reporting Summary

Nature Portfolio wishes to improve the reproducibility of the work that we publish. This form provides structure for consistency and transparency in reporting. For further information on Nature Portfolio policies, see our Editorial Policies and the Editorial Policy Checklist.

Statistics

For all statistical analyses, confirm that the following items are present in the figure legend, table legend, main text, or Methods section.

n/a	Confirmed	
☐	☒	The exact sample size (*n*) for each experimental group/condition, given as a discrete number and unit of measurement
☒	☐	A statement on whether measurements were taken from distinct samples or whether the same sample was measured repeatedly
☐	☒	The statistical test(s) used AND whether they are one- or two-sided<br>*Only common tests should be described solely by name; describe more complex techniques in the Methods section.*
☐	☒	A description of all covariates tested
☐	☒	A description of any assumptions or corrections, such as tests of normality and adjustment for multiple comparisons
☐	☒	A full description of the statistical parameters including central tendency (e.g. means) or other basic estimates (e.g. regression coefficient) AND variation (e.g. standard deviation) or associated estimates of uncertainty (e.g. confidence intervals)
☐	☒	For null hypothesis testing, the test statistic (e.g. *F*, *t*, *r*) with confidence intervals, effect sizes, degrees of freedom and *P* value noted<br>*Give P values as exact values whenever suitable.*
☐	☒	For Bayesian analysis, information on the choice of priors and Markov chain Monte Carlo settings
☐	☒	For hierarchical and complex designs, identification of the appropriate level for tests and full reporting of outcomes
☒	☐	Estimates of effect sizes (e.g. Cohen's *d*, Pearson's *r*), indicating how they were calculated

Our web collection on statistics for biologists contains articles on many of the points above.

Software and code

Policy information about availability of computer code

Data collection	Software and code was used to download data from the National Centre of Environmental Predictions (NCEP), using the curl package (version 5.0.0). Other data were not collected using software or code.

Data analysis	All code is available at https://github.com/p-pottier/Vulnerability_amphibians_global_warming (accessible with the webpage: https://p-pottier.github.io/Vulnerability_amphibians_global_warming/).<br>All data analyses were performed using R statistical software (version 4.3.0). The following packages were used for processing and/or analysing the data: RNCEP (version 1.0.10), terra (version 1.7-46), emmeans (version 1.7.3), optimx (version 2023-10.21), ggeffects (version 1.2.2), cowplot (version 1.1.1), lwgeom (version 0.2-13), ggspatial (version 1.1.8), metafor (version 4.2-0), numDeriv (version 2016.8-1.1), metadat (version 1.2-0), rnaturalearthhires (version 0.2.1), rnaturalearthdata (version 0.1.0), rnaturalearth (version 0.3.3), futile.logger (version 1.4.3), future.apply (version 1.10.0), furrr (version 0.3.1), future (version 1.33.0), rlang (version 1.1.1), gamm4 (version 0.2-6), lme4 (version 1.1-33), mgcv (version 1.8-40), nlme (version 3.1-157), MCMCglmm (version 2.34), coda (version 0.19-4), Matrix (version 1.5-4), microclima (version 0.1.0), NicheMapR (version 3.3.2), RNetCDF (version 2.6-2), data.table (version 1.14.8), sf (version 1.0-14), zoo (version 1.8-12), curl (version 5.0.0), abind (version 1.4-5), doParallel (version 1.0.17), iterators (version 1.0.14), foreach (version 1.5.2), rgdal (version 1.6-7), taxize (version 0.9.100), rredlist (version 0.7.1), letsR (version 4.0), rgeos (version 0.6-2), rasterSp (version 0.0.1), raster (version 3.6-23), sp (version 2.0-0), ggbeeswarm (version 0.7.2), ggExtra (version 0.10.0), here (version 1.0.1), ggstatsplot (version 0.11.1), ggdist (version 3.2.1), RColorBrewer (version 1.1-3), ggnewscale (version 0.4.10.9000), tidytree (version 0.4.2), phytools (version 1.5-1), ggtreeExtra (version 1.7.0), ggtree (version 3.5.0.901), R.utils (version 2.12.2), R.oo (version 1.25.0), R.methodsS3 (version 1.8.2), patchwork (version 1.2.0.9000), naniar (version 1.0.0), ape (version 5.7-1), maps (version 3.4.1), viridis (version 0.6.4), viridisLite (version 0.4.2), kableExtra (version 1.3.4), lubridate (version 1.9.2), forcats (version 1.0.0), stringr (version 1.5.0), dplyr (version 1.1.2), purrr (version 1.0.1), readr (version 2.1.4), tidyr (version 1.3.0), tibble (version 3.2.1), ggplot2 (version 3.5.1), and tidyverse (version 2.0.0).

For manuscripts utilizing custom algorithms or software that are central to the research but not yet described in published literature, software must be made available to editors and reviewers. We strongly encourage code deposition in a community repository (e.g. GitHub). See the Nature Portfolio guidelines for submitting code & software for further information.

Data

Policy information about availability of data

All manuscripts must include a data availability statement. This statement should provide the following information, where applicable:

- Accession codes, unique identifiers, or web links for publicly available datasets
- A description of any restrictions on data availability
- For clinical datasets or third party data, please ensure that the statement adheres to our policy

All heat tolerance data were compiled in a previous study (Pottier et al. 2022. Scientific Data), climatic data were taken from the National Center for Environmental Predictions (NCEP) and TerraClimate, species distribution ranges were taken from the International Union for the Conservation of Nature (IUCN) red list, ecotype and body mass data were taken from Wu et al. (2024, EcoEvoRxiv), Johnson et al. (2023, Global Ecology and Biogeography), and Santini et al. (2018, Integrative Zoology), and phylogenetic data were taken from Jetz & Pyron (2018, Nature Ecology & Evolution). All data sources are acknowledged and referenced in the manuscript.

Raw and processed data are available at https://github.com/p-pottier/Vulnerability_amphibians_global_warming, and are archived in Zenodo (https://doi.org/10.5281/zenodo.14498866). Note, however, that some intermediate data files were too large to be shared online. These files are available upon request. TerraClimate data is available from https://www.climatologylab.org/terraclimate.html and NCEP data is available from https://psl.noaa.gov/thredds/catalog/Datasets/ncep.reanalysis2/gaussian_grid/catalog.html.

Research involving human participants, their data, or biological material

Policy information about studies with human participants or human data. See also policy information about sex, gender (identity/presentation), and sexual orientation and race, ethnicity and racism.

Reporting on sex and gender	NA
Reporting on race, ethnicity, or other socially relevant groupings	NA
Population characteristics	NA
Recruitment	NA
Ethics oversight	NA

Note that full information on the approval of the study protocol must also be provided in the manuscript.

Field-specific reporting

Please select the one below that is the best fit for your research. If you are not sure, read the appropriate sections before making your selection.

☐ Life sciences ☐ Behavioural & social sciences ☒ Ecological, evolutionary & environmental sciences

For a reference copy of the document with all sections, see nature.com/documents/nr-reporting-summary-flat.pdf

Ecological, evolutionary & environmental sciences study design

All studies must disclose on these points even when the disclosure is negative.

Study description	In this study, we assessed the global vulnerability of amphibians to extreme heat events in different climatic scenarios and thermal refugia. We developed a new approach to solve taxonomical and geographical biases in thermal limits using Bayesian phylogenic data imputation. We then integrated predicted thermal limits with body temperatures estimated from biophysical models to quantify the proximity of heat tolerance limits to field body temperatures experienced in shaded microhabitats.
Research sample	All heat tolerance data were compiled in a previous study (Pottier et al. 2022. Scientific Data), climatic data were taken from the National Center for Environmental Predictions (NCEP) and TerraClimate, species distribution ranges were taken from the International Union for the Conservation of Nature (IUCN) red list, ecotype and body mass data were taken from Wu et al. (2024, in prep), Johnson et al. (2023, Global Ecology and Biogeography), and Santini et al. (2018, Integrative Zoology), and phylogenetic data were taken from Jetz & Pyron (2018, Nature Ecology & Evolution). All data sources are acknowledged and referenced in the manuscript. Heat tolerance data were filtered to 2,661 estimates from 524 species using predefined inclusion criteria, and our data imputation procedure has expanded this sample to data from 5203 species (spanning up to 204,808 populations for each microhabitat and climatic scenario). Detailed sample sizes are provided in the Results section.
Sampling strategy	Heat tolerance data were only included if they were measured using a dynamic methodology, if the acclimation temperature was recorded, if the species was listed in the phylogeny from Jetz & Pyron (2018, Nature Ecology & Evolution), and if their geographical range was reported in the IUCN red list.
Data collection	PPottier, NCW, PPollo and ANRV collected all data. Heat tolerance data were compiled by PPottier, PPollo and ANRV, climatic data, distribution ranges, and phylogenetic data were compiled by PPottier, and ecotype and body mass data were compiled by PPottier, NCW, PPollo, and ANRV.
Timing and spatial scale	Heat tolerance data was collected up to May 2021, and cover a large spatial scale. Geographic biases are discussed in the manuscript and resolved using data imputation.
Data exclusions	Data not matching our inclusion criteria (i.e., heat tolerance data were only included if they were measured using a dynamic methodology, if the acclimation temperature was recorded, if the species was listed in the phylogeny from Jetz & Pyron (2018, Nature Ecology & Evolution), and if their geographical range was reported in the IUCN red list) were excluded.
Reproducibility	All analyses are reproducible using the code provided. We also provided a rendered html file to walk the reader through the analyses and facilitate reproducibility.
Randomization	No randomization was involved in this study.
Blinding	No blinding was involved in this study.

Did the study involve field work? ☐ Yes ☒ No

Reporting for specific materials, systems and methods

We require information from authors about some types of materials, experimental systems and methods used in many studies. Here, indicate whether each material, system or method listed is relevant to your study. If you are not sure if a list item applies to your research, read the appropriate section before selecting a response.

Materials & experimental systems

n/a	Involved in the study
☒	☐ Antibodies
☒	☐ Eukaryotic cell lines
☒	☐ Palaeontology and archaeology
☒	☐ Animals and other organisms
☒	☐ Clinical data
☒	☐ Dual use research of concern
☒	☐ Plants

Methods

n/a	Involved in the study
☒	☐ ChIP-seq
☒	☐ Flow cytometry
☒	☐ MRI-based neuroimaging

Plants

Seed stocks	*Report on the source of all seed stocks or other plant material used. If applicable, state the seed stock centre and catalogue number. If plant specimens were collected from the field, describe the collection location, date and sampling procedures.*
Novel plant genotypes	*Describe the methods by which all novel plant genotypes were produced. This includes those generated by transgenic approaches, gene editing, chemical/radiation-based mutagenesis and hybridization. For transgenic lines, describe the transformation method, the number of independent lines analyzed and the generation upon which experiments were performed. For gene-edited lines, describe the editor used, the endogenous sequence targeted for editing, the targeting guide RNA sequence (if applicable) and how the editor was applied.*
Authentication	*Describe any authentication procedures for each seed stock used or novel genotype generated. Describe any experiments used to assess the effect of a mutation and, where applicable, how potential secondary effects (e.g. second site T-DNA insertions, mosiacism, off-target gene editing) were examined.*
